# Knowledge, Attitude, and Practice with Respect to Antibiotic Use among Chinese Medical Students: A Multicentre Cross-Sectional Study

**DOI:** 10.3390/ijerph15061165

**Published:** 2018-06-04

**Authors:** Yanhong Hu, Xiaomin Wang, Joseph D. Tucker, Paul Little, Michael Moore, Keiji Fukuda, Xudong Zhou

**Affiliations:** 1 School of Public Health, The University of Hong Kong, Hong Kong, China; huhubest@gmail.com (Y.H.); kfukuda@hku.hk (K.F.); 2 School of Public Health, Zhejiang University, Hangzhou 310058, China; xiaominzjuhvd@gmail.com; 3 UNC-China Project, University of North Carolina at Chapel Hill, Chapel Hill, NC 27599, USA; jdtucker@med.unc.edu; 4 Primary Care and Population Science, University of Southampton, Southampton SO16 5ST, UK; p.little@soton.ac.uk (P.L.); mvm198@soton.ac.uk (M.M.)

**Keywords:** antibiotic use, knowledge and attitude, medical students, multicentre

## Abstract

Objective: Inappropriate antibiotic use leads to antibiotic resistance. This has become a serious global crisis, with more multi-drug resistant infections and fewer effective antibiotics available. This study aims to understand knowledge, attitude, and practice (KAP) with respect to antibiotic use for self-limiting illnesses among medical students in China. Methods: An online cross-sectional survey instrument questionnaire was distributed in six regional universities in China from September to November 2015. Overall, 1819 medical students were enrolled. A pre-tested questionnaire was delivered by the researchers. KAP scores were calculated to determine the appropriation. Chi-squared and multivariable logistic regression and adjusted odd ratios (aORs) with 95% confidence interval (CI) were used to assess the relationship between the demographic characteristics and antibiotic use knowledge and behaviour. Results: In total, 11,192 students completed the questionnaires, with a response rate of 95%. In total, 529 (29%) medical students reported at least one self-limiting illness in the prior month. Of those with a self-limiting illness, 285 (54%) self-medicated, with 77 (27%) using antibiotics; 111 (21%) went to see a doctor, of which 64 (58%) were prescribed antibiotics, and 133 did nothing (25%). In the past year, 279 (15%) of medical students had used antibiotics as prophylaxis, and 273 (15%) of medical students had demanded an antibiotic from a doctor. Meanwhile, 1166 (64%) of them kept a personal stock of antibiotics, and 1034 (57%) of them had bought antibiotics at a pharmacy, of which 97% were purchased without a prescription. Students with high KAP scores with respect to antibiotics were significantly less likely to self-medicate with antibiotics (aOR 0.37, 95% CI 0.15–0.91, *p* = 0.031), use antibiotics for prophylaxis (aOR 0.35, 95% CI 0.21–0.60, *p* < 0.0001), or demand an antibiotic (aOR 0.46, 95% CI 0.26–0.81, *p* = 0.007) from the doctor. Logistical regression showed that students whose fathers had a higher education level, whose mothers had medical background, who were from urban areas were more likely to stock antibiotics and self-medicate with antibiotics. Conclusion: High rates of antibiotic self-medication for self-limiting illness and stocking of antibiotics among medical students were observed. Along with the high rates of medical students receiving unnecessary antibiotics from their doctors were observed. The students’ knowledge and attitude towards to antibiotics, which drive prescribing, highlight the urgent need for effective antibiotic stewardship and training programs in Chinese healthcare institutes and medical schools.

## 1. Introduction

Antimicrobial resistance (AMR) [1] is an urgent public health crisis [2,3,4]. Unnecessary antibiotic use increases the risk of AMR and reduces the efficacy of antibiotics needed for treating bacterial infection. China has the second largest antibiotic consumption in the world and very high rates of unnecessary antibiotic use both in humans and animals [5]. A systematic review showed that more than 80% of Chinese outpatients with upper respiratory infections were prescribed antibiotics [6]. This is associated with increasingly high rates of AMR in hospitals and the environment. Perhaps consequently, increasing trends of AMR have been observed in the yearly national surveillance report in many developing countries such as China [7]. China is at particular risk of AMR given the large population in China and increasing rates of international travel. Recent research has found colistin resistance genes among 16 community patients in China [8], indicating the urgent need for control of AMR.

The Chinese national antibiotic stewardship program started in 2012, with a primary focus on the medication supply chain, financial compensation for medicines from the government, and training of healthcare providers [9]. However, the public campaign has few involved clinicians and patient-oriented interventions. Self-medication of patients with antibiotics is also an important contributor to antibiotic misuse, particularly considering the easy access to antibiotics from pharmacies in many developing countries [10]. Antibiotics from pharmacies can only be sold by prescription, as per restrictions imposed by the China Food and Drug Administration in 2004. Despite this measure, it is still common to obtain antibiotics from pharmacies without a prescription [11]. A recent study with an on-line survey in a university in eastern China showed that 48% of university students had used over-the-counter antibiotics [12].

High rates of antibiotic misuse may arise from the knowledge and behaviours of doctors and the pressure they receive from patients [13]. In recent years, the relationship between Chinese doctors and patients has deteriorated [14]. Past standards of routine clinical practice might also have influenced public perceptions and led to inaccurate understandings about appropriate antibiotic use [15]. Medical students are particularly important since they will be the future leaders in clinic practice, responsible for antibiotic prescription and communication about antibiotic use with their patients. Several studies have described medical students’ antibiotic related behaviours in Nepal [10], Pakistan [16], Europe [17,18,19], and the USA [20]. The self-medication rate ranged from 30 to 80%, and antibiotic self-medication rates ranged from 19 to 100% [10,12,13]. However, there has been no recent substantial study on medical students’ antibiotic use behaviour in China, only with university students in general [12,21,22]. One study included a single university, where the antibiotic use rate was higher in non-medical students than in medical students, and antibiotic use in self-limiting illnesses ranged from 15 to 50% [22]. For this study, the main objective is to understand the knowledge, attitude, and practice (KAP) with respect to antibiotic use for self-limiting illnesses among Chinese medical students, This study will help to develop an intervention of training curriculum in universities in the future after this survey [23], which will help to inform policy guidance and interventions to improve their training on appropriate antibiotic use that should lead to changes in behaviour.

## 2. Methods

### 2.1. Participants

This study was conducted in six universities in six regions of China. It was a cross-sectional survey of antibiotic-related knowledge and behaviours of university students. A cluster randomized sampling method was adopted. A detailed description of sampling has been described elsewhere [24]. Six high-ranking comprehensive universities were selected: the Nankai, Zhejiang, Jilin, Lanzhou Wuhan, and Guizhou Universities. The KAP theory-based questionnaire was developed and adapted through a literature review from the Centers for Disease Control (CDC) in the United States and China [21,22,25]. This study aimed to use KAP to understand Chinese medical students’ antibiotic use beliefs and behaviours, in order to explore the determining factors for antibiotic use behaviours and inform an effective intervention to reduce unnecessary antibiotic use among medical students. The survey was conducted from September to November 2015 (Ethics approval: Zhejiang University Ethic committee, reference number: ZGL20160922).

### 2.2. Questionnaire

The questionnaire comprised four constructs of the KAP regarding antibiotic use for self-limiting illnesses and syndromes, including the common cold, fever, sore throat, headache, ear pain, diarrhoea, and abdominal pain. For each item, the response was yes/no or unknown/uncertain. The behavioural outcomes of interest were self-medication with antibiotics in the past month for self-limiting illness; demanding an antibiotic from a clinician; stocking of a supply of antibiotics at home; and the use of antibiotics to prevent common cold in the past year. Students were asked to state the chemical or brand names of antibiotics they had used. The detailed questionnaire is provided in Table 1.

### 2.3. Data Collection

Data collection was a part of a large cross-sectional study of Chinese university students of science, social science and humanity, and medicine [26,27]. The online survey Wen Juan Xing (https://www.wjx.cn/) was conducted using smartphones. A gratuity of RMB3 (US$0.5 in 2015) was paid via smartphone to all students who completed the questionnaire. Details of the method have been described elsewhere [27]. For the current study we only selected the medical students for analysis.

### 2.4. Statistical Analysis

A KAP score for antibiotic-related knowledge was created. There are 15 questions on knowledge and attitude. Each question represents one score. A score of 0 to 7 was categorized as a low level of knowledge, 8 to 12 as medium, and 13 to 15 as high. As students would have practical rotations in clinics during their studies, we divided them into pre-clinical (Uy1–Uy3) and post clinical medical students (Uy4–Uy8). χ^2^ test was used to examine associations between the antibiotic-related knowledge score and behaviours. Binary logistic regression was used to control for the socio-demographic variables. Analyses were done with Stata14 (Statacorp LLC, College Station, TX, USA).

## 3. Results

A total of 1819 medical students across the six universities completed all key items of the questionnaire, while 62 (5%) questionnaires were discarded because of non-completion of key variables.

### 3.1. Socio-Demographic Characteristics 

Of the 1819 medical students 64% were female and aged from 16 to 40 (mean 22 (±1.5)). Rural and urban residents were equally represented. The majority were undergraduate students (diploma and bachelor’s degree; 68%). Most the students (86%) came from low-income families (less than RMB10,000 per month = US$1538 per month) (Table 2) Most of the students’ parents had limited or no college education and with no medical background. For knowledge and attitudes, more than 85% of the medical students were aware of overuse of antibiotics in China as a serious problem that could cause AMR in the future and lead to difficulties in treating bacterial infections. More than 60% of the medical students answered correctly that the common cold was a self-limiting disease not requiring antibiotics. Ninety-two percent of medical students agreed that antibiotics were effective for treating bacterial infections. However, only 47% of medical students did not agree that antibiotics could reduce the symptoms of the common cold. In total, five behaviour outcomes were included in this study: self-medication (SM), self-medication with antibiotics (SMA), stocking of antibiotics in the dormitory/at home (stocking) [3], demanding antibiotics from a doctor (demand), and the use of an antibiotic for prophylaxis. There were 85 (4.7%) students that had KAP scores of 0–7, 999 (54.9%) with KAP scores of 8–12, and 735 (40.4%) with KAP scores of 13–15 (Table 2).

### 3.2. Antibiotic Use Behaviours for Self-Limiting Illness and Symptoms 

**Figure 1:**
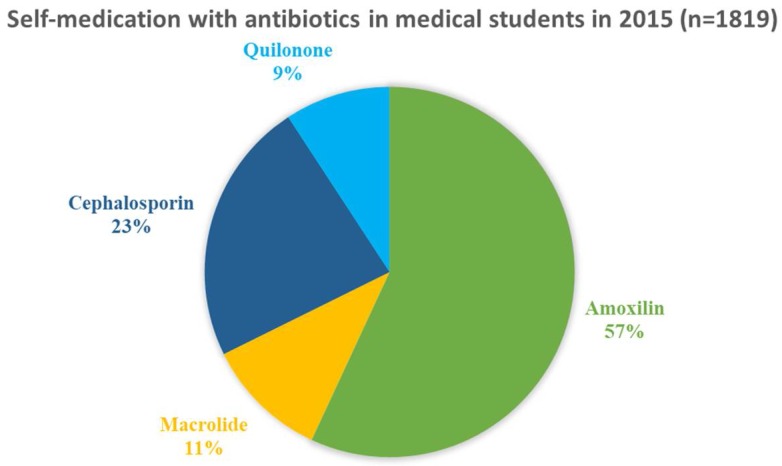
Self-medication with antibiotics in Chinese medical students in the past month in 2015 (*n* = 1819).

In the past month, 529 (29.1%) medical students reported that they had experienced at least one self-limiting illness or syndrome. Of them 59.2% had a common cold, 58.0% had a headache, 57.1% had a sore throat, 52.6% had diarrhoea, 53.2% had abdominal pain, 45.8% had a fever, and 25.0% had suspected pneumonia. Some medical students reported overlapping symptoms. Of the students reporting illness of cold, 61 (16.8%) went to see a doctor and 215 (59.2%) of them were self-medicated and 87 (24.0%) of them with no specific treatment. Of those who went to see a doctor with prescribed antibiotics, 25 (39.0%) were given injectable antibiotics. Over half (285 or 54.0%) of the medical students who reported illness treated themselves for their symptoms (see Table 3). Of the self-treating students 77 (27.0%) used antibiotics, the most frequency were: 37 (56.9%) used amoxicillin, 15 (23.1%) cephalosporin, 7 (10.8%) macrolides, and 6 (9.2%) used quinolone antibiotics (Figure 1). 

### 3.3. Determinants of Antibiotic Use Behaviours 

Medical students from different universities had different antibiotic use behaviours. Students from Nankai University had the highest rates of SM and stocking but the lowest rates for demand for antibiotics and use for prophylaxis. Lanzhou University students had the highest rates of SMA and demand for antibiotics. Guizhou had the highest rates for antibiotic use for prophylaxis (Table 4).

Older and female students had significantly higher rates of stocking antibiotics. There were no significant differences in behaviours when analysed by study year or degree level. Students who had a father with medical background had higher rates of SM and stocking of antibiotics. Also, students who had a mother with medical background had higher rates of SM, SMA, and stocking. Higher education levels for both parents were associated with higher rates of SM, SMA, and stocking. Higher household monthly income (>US$1538) students had higher rates of SM and SMA but a lower rate of prophylaxis. Students from urban areas had higher rates of SM, SMA, and stocking, and lower rates of prophylaxis. Students with lower KAP scores had higher rates of demand and prophylaxis.

### 3.4. Factors Influencing Antibiotic Use Behaviours 

After adjusting for university, age, sex, education level, study year, family income, home town, parents’ educational level, and whether parents with medical background, it was found that medical students with the highest KAP scores were less likely to demand antibiotics (aOR 0.46 95% CI 0.26–0.81, *p* = 0.007), SMA (aOR 0.37 95% CI 0.15–0.91, *p* = 0.031), and use antibiotics for prophylaxis (aOR 0.35 95% CI 0.21–0.60, *p* < 0.001). Those aged between 21 and 30 years old were more likely to demand antibiotics from a doctor. Female students were more likely to stock antibiotics. Students whose fathers had a higher educational level, whose mothers with medical backgrounds, and whose hometowns were urban were more likely to SMA and stock antibiotics. Students whose hometowns were rural were more likely to use antibiotics for prophylaxis (Table 5).

## 4. Discussion

### 4.1. Summary of the Study

This is the first nationwide survey to explore the knowledge and behaviour regarding antibiotic use among Chinese medical students. This study has shown that medical students have high rates of stocking antibiotics and self-medication. One in four used unnecessary antibiotics for a self-limiting illness, one in seven used antibiotics for preventing a cold, and one in seven demanded antibiotics from a doctor. Also, students that had a mother with medical background were more likely to self-medicate and stock antibiotics.

### 4.2. Comparison with the Existing Studies

Unnecessary antibiotic use among medical students seems to be a worldwide phenomenon which is likely to contribute to the risk of AMR, adverse events, and health economic burden. In the current study, 27% of the medical students reported SMA. This rate is much higher than in the studies found in Australia [28] and lower than in Kosovo [18]. Even higher SMA rates were observed in Italian medical students (45%) [17]. Another study showed high rates (60%) of SMA among Pakistani pharmacy students [29]. Rates among university students are in general high in several other studies from low- and middle-income countries [10,30,31]. Our study may have lower risk for recall bias as this study asked about the illness in the past month, while many of the other studies focused on the past 12 months or life-time illness, which might underestimate the actual rates. A study from Zhu and colleagues showed the SMA rate was 74% among Chinese university students [12]. This is three times higher than our study, although these were all undergraduate students without reference to the course of study. Medical students might be expected to have lower rates of inappropriate antibiotic use as they have received some medical training regarding bacteria and antibiotics.

In the current study, the antibiotic stocking rate (64%) is higher than the study from a western university in China (56%) [21]. The frequency of SMA for symptom relief, using antibiotics for prophylaxis, and demanding antibiotics might be higher than the reported number in this study; participants may tend to not to report these behaviours, as many medical students know this is inappropriate and may have engaged in social desirability responding.. The persistently high rates demonstrate the need for further training about consequences of unnecessary antibiotic use for medical students.

More than half of the medical students who went to see a doctor received antibiotics during their consultation for a self-limiting illness, of which 39% received injectable antibiotics. Three-quarters of the students who received these antibiotics considered that they were necessary, even though 4 in 5 affirmed that colds are self-limiting and resolve themselves without treatment. The higher rate of knowledge does not always predict actual prudent behaviour [32]. The students’ belief that antibiotic use was necessary may derive from uncertainty about the nature of their illness. There may have been some students who used the same antibiotic twice or even more frequently in the previous year and reported this as only “1” in our survey. This may underestimate the real rate of SMA. In total, 15% of the students had demanded an antibiotic from a doctor in the previous year, and 15% of them had used antibiotics for prophylaxis. It is perhaps not surprising that one of the major reasons that doctors prescribe is because of pressure due to patient demand [33,34], which is likely to be even more common when the patient is a medical student.

In the regression analysis, a low KAP score was associated with a greater likelihood of SMA, demand, and prophylaxis. It is to be expected that some students are not aware of the implications of AMR if they lack the appropriate knowledge of how a common cold should be treated. In addition, these students perceive a large benefit from antibiotics, and there are low barriers to antibiotic use. The higher KAP scores may reflect easy access to antibiotics either because of proximity or economics, which may explain why students from urban areas are more likely to self-medicate with and store antibiotics. There are more pharmacies in urban areas than in rural areas [11]. Furthermore, in China pharmacies are loosely regulated, and people can buy antibiotics without a prescription. In our study, 97% of the students reported that they had bought antibiotics without a prescription. Female students were more likely to stock antibiotics than male students, perhaps reflecting a natural tendency of females to prepare for sickness in advance. This has been found in other Chinese studies showing a female predisposition to stock medications [12,35]. The gender association may also be reflected in the higher proportion of students whose mothers were doctors stocking and self-medicating with antibiotics. While having a parent with medical background would be expected to increase access to antibiotics, although there was a trend, the association of having a father with medical background was not significant. One might expect that having a parent with medical background would reduce unnecessary antibiotic use, however knowledge of appropriate antibiotic use is not always reflected in physician behaviour [36], perhaps because inappropriate prescribing of antibiotics is even more common in older generations.

### 4.3. Implications of This Study

This study has several implications for the education of Chinese medical students or early-stage medical professionals regarding antibiotic stewardship in China. The KAP scores were generally high, nevertheless the KAP scores were strongly associated with antibiotic use behaviours, so there is clearly still a knowledge gap for many medical students regarding the use of unnecessary antibiotics for self-limiting illnesses. This is consistent with the current studies with high antibiotic prescribing rates in primary care settings in China [36,37]. Medical practitioners may also prescribe because they are uncertain how to distinguish between a self-limiting viral illness and a serious bacterial illness and need the confidence to treat each appropriately. Reinforcing the antibiotic stewardship program using effective methods is urgently needed in the health system and undergraduate medical education. In several medical schools like Johns Hopkins, Brown University and other U.S universities, current infectious disease fellows have to complete an antibiotic stewardship program during the fellowship training, which is a compulsory course for them [38]. Such programmes could be modified for use in China and other developing countries.

Medications to manage symptoms should be recommended from the supply and consumer chains. It is hard to believe that there was only one student who used antipyretic medication to relieve the symptoms, so training in the use of symptomatic medication is warranted. Currently, it is easy to access the antibiotics from pharmacy stores in China without a prescription, and since doctors’ salaries are related to prescriptions [11], there are also perverse incentives to prescribe antibiotics. Regulations for pharmacies, particularly involving pharmacists and both public and private pharmacy stores into the stewardship program are clearly important.

Patient demand is also part of the drive for unnecessary antibiotic use, as shown in this survey, and supported by other studies [39,40,41]. Medical students should be taught specifically how to communicate with patients about appropriate antibiotic use and how to negotiate requests from patients for antibiotics, as well as policies on the reinforcing the existing monitoring system. It is only through changing beliefs and behaviours of patients, doctors, and pharmacists that unsupervised pharmacy purchases of antibiotics and other medicines can be reduced in the absence of major changes in standard pharmacy practice.

### 4.4. Limitations of This Study

This study has some limitations. This is a cross-sectional study, thus it is difficult to ascribe causal associations. Recall bias is also possible but as medical students were young and the recall period was only the prior month and the prior year, this bias is probably limited. Although the online methodology should have minimized social desirability bias, this bias would tend to lead to an underestimation of the reported rates, and this can be inferred indirectly given the high scores on the perceived severity and low scores on the perceived benefits and barriers. Since this was an online questionnaire through smartphones, looking up the correct answer may have over-estimated the knowledge scores. However, the questionnaire answers were completed within 15 min, there was little time for the students to search for answers online, and would only affect knowledge but not behaviour. We did not explore in depth other alternative therapies students might have used instead of antibiotics. We may have underestimated the extent of prophylactic use in the prior 12 months since one person might have 2–4 episodes in a year [42]. The presence of researchers ensured that the response rate was very high compared to recent online surveys among students. We therefore believe that our survey is reasonably representative of the student population of high-level universities in China.

## 5. Conclusions

Antibiotic use among Chinese medical students for self-limiting illness is common, and reflects antibiotic use behaviour in the public. National education programs for strengthening public knowledge and awareness of appropriate antibiotic use are urgently needed and should be reinforced in medical education and outpatient settings with multifaceted components to reduce unnecessary antibiotic use. Appropriate antibiotic use policies involving pharmacy regulations is also urgently needed.

## Figures and Tables

**Table 1:** Questionnaire details were based on knowledge, attitude, and practice (KAP) constructs in the survey among medical students in China in 2015.

Constructs	Questionnaire (1) True (2) False (3) Do not know
Knowledge	1. Antibiotics are effective for viral infections 2. Antibiotics have the same effects as anti-inflammatory drugs 3. If one needs to use antibiotics, it is best to give them by intravenous infusion 4. Once the symptoms are relieved, one should immediately stop using antibiotics 5. We will have few antibiotics to use in the future if we do not use antibiotics properly 6. The more frequently people use antibiotics, the more difficult it will be to treat bacterial infections 7. Antibiotics are effective for treating common cold (cough, runny nose) 8. Antibiotics can speed up recovery from flu 9. Antibiotics can relieve the symptoms of colds 10. Antibiotics are effective for sore throats 11. One needs to take antibiotics for a cold with green mucus 12. Antibiotics are effective for treating the common cold 13. Overseas antibiotics are more effective than domestic ones
Attitude	(1) Yes (2) No (3) Unknown/Uncertain 14. Do you think antibiotic overuse is a serious problem in China? 15. Do you prefer to use antibiotics when you: 15.1. have sore throat? 15.2. have a cough? 15.3. have a runny nose? 15.4. have a common cold? 15.5. have a fever? 15.6. have diarrhoea?
Practice	16.1. What illness/symptoms have you had in the last month? (a) sore throat; (b) cough; (c) runny nose; (d) common cold; (e) fever; (f) diarrhoea; (g) headache; (h) abdominal pain; (i) suspected pneumonia; (j) other/s please write down_____ 16.2. What have you done for an illness you have experienced in the last month? (1) self-medicated (2) seen a doctor (3) nothing 16.3. If you saw a doctor, did you receive antibiotic prescription? 16.3.1. If you received antibiotic prescription, which kind of antibiotic did you receive? (1) Oral (2) intravenous infusion 17. If you self-medicated, did you use antibiotics? (1) yes (2) no (3) do not remember 17.1. If yes, which antibiotic did you use? 18. Are there any left-over antibiotics at your home/dormitory (not for current use)? (1) yes (2) no (3) do not remember 19. Did you ask for an antibiotic if you did not receive one from a clinician during the consultation? (1) yes (2) no (3) do not remember 20. Did you take antibiotics to prevent diseases (such as common cold) in the past year? (1) yes (2) no (3) do not remember

**Table 2:** Socio-demographic characteristics of university medical students in China in 2015 (*n* = 1819).

Characteristics	*N* (%)
University (Province)	
Nankai University (Tianjin)	281 (15.5%)
Zhejiang University (Zhejiang)	302 (16.6%)
Jilin University (Jilin)	341 (18.8%)
Wuhan University (Hubei)	303 (16.7%)
Lanzhou University (Gansu)	292 (16.1%)
Guizhou University (Guizhou)	300 (16.5%)
Gender	
Male	661 (36.3%)
Female	1158 (63.7%)
Age (years)	
16–20	717 (39.4%)
21–25	980 (53.9%)
26–40	122 (6.71%)
Education level	
Diploma-B. A	1244 (68.4%)
Master–PhD	575 (31.6%)
Study year	
Uy1–Uy3/pre clinic	779 (42.8%)
Uy4–Uy8/post clinic	1040(57.2%)
Father’s education	
Less than college level	1341 (73.4%)
Above college level	478 (26.3%)
Mother’s education	
Less than college level	1448 (79.6%)
Above college level	3718 (20.4%)
Medical background father	
Yes	106 (5.8%)
No	1713 (94.2%)
Medical background mother	
Yes	124 (6.8%)
No	1695 (93.2%)
Household income/month	
< US$1538	1565 (86.0%)
=/> US$1538	254 (14.0%)
Hometown	
Rural	916 (50.4%)
Urban	903 (49.6%)

**Table 3:** Antibiotic use behaviour for self-limiting illness symptoms among medical students in China in 2015 (*n* = 1819).

Symptoms	Cases (*n*)	SM	SMA	See a Doctor	Antibiotic Prescribed	Injectable Antibiotics	With no Specific Treatment
Cold	363	215 (59.2%)	66 (30.7%)	61 (16.8%)	40 (65.6%)	14 (35.0%)	87 (24.0%)
Fever	72	33 (45.8%)	11 (33.3%)	25 (34.7%)	20 (80.0%)	8 (40.0%)	14 (19.4%)
Sore throat	182	104 (57.1%)	36 (34.6%)	36 (19.8%)	27 (75.0%)	11 (40.7%)	42 (23.1%)
Ear pain	29	9 (31.0%)	5 (55.6%)	15 (51.7%)	11 (73.3%)	4 (36.4%)	5 (17.2%)
Headache	81	47 (58.0%)	12 (25.5%)	18 (22.2%)	14 (77.8%)	6 (42.9%)	16 (19.8%)
Flu-like illness	23	9 (39.1%)	5 (55.6%)	15 (65.2%)	8 (53.3%)	4 (50.0%)	5 (21.7%)
Diarrhoea	95	50 (52.6%)	16 (32.0%)	17 (17.9%)	10 (58.8%)	2 (20.0%)	28 (29.5%)
Suspected pneumonia	8	2 (25.0%)	1 (50.0%)	6 (75.0%)	4 (66.7%)	1 (25.0%)	0 (0%)
Abdominal pain	47	25 (53.2%)	4 (16.0%)	7 (14.9%)	5 (71.4%)	1 (20.0%)	15 (31.9%)

Note: SM: Self-medication; SMA: Self-medication with antibiotics; Students may have overlapping symptoms; the total number of students who reported having illness in the past month was 529.

**Table 4:** Determining factors related to antibiotic use behaviours among medical students in China in 2015.

	SM	*p*	SMA	*p*	Stock	*p*	Demand	*p*	Prophylaxis	*p*
Universities		0.001		0.082		0.010		0.006		0.001
Lanzhou	59 (20.2%)		20 (6.9%)		191 (65.4%)		55 (18.8%)		58 (19.9%)	
Nankai	62 (22.1%)		15 (5.3%)		201 (71.5%)		29 (10.3%)		29 (10.3%)	
Jilin	45 (13.2%)		14 (4.1%)		215 (63.1%)		61 (17.9%)		53 (15.5%)	
Wuhan	33 (10.9%)		9 (3.0%)		171 (56.4%)		33 (10.9%)		33 (10.9%)	
Zhejiang	44 (14.6%)		7 (2.3%)		192 (63.6%)		42 (13.9%)		44 (14.6%)	
Guizhou	42 (14.0%)		12 (4.2%)		196 (65.3%)		53 (17.7%)		62 (20.7%)	
Age (years)		0.947		0.440		0.002		0.061		0.097
16–20	114 (15.9%)		35 (4.9%)		464 (64.7%)		90 (12.6%)		125 (17.4%)	
21–25	153 (15.6%)		36 (3.7%)		607 (61.9%)		163(16.6%)		140 (14.3%)	
26–40	18 (14.8%)		6 (4.9%)		95 (77.9%)		20 (16.4%)		14 (11.5%)	
Sex		0.455		0.470		0.021		0.662		0.065
Male	98 (14.8%)		25 (3.8%)		401 (60.7%)		96 (14.5%)		115 (17.4%)	
Female	187 (16.2%)		52 (4.5%)		765 (66.1%)		177 (15.3%)		164 (14.2%)	
Study year		0.327		0.654		0.412		0.001		0.101
Uy1–Uy3	78 (16.8%)		20 (2.5%)		494 (63.4%)		40 (14.7%)		147 (18.9%)	
Uy4–Uy8	112 (19.5%)		38 (3.4%)		290 (62.4%)		532 (68.3.%)		68 (14.1%)	
Education level		0.480		0.277		0.158		0.131		0.016
Diploma-B. A	200 (16.1%)		57 (4.6%)		784 (63.0%)		176 (14.2%)		208 (16.7%)	
Master/PhD	85(14.8%)		20 (3.5%)		382 (66.4%)		97 (16.9%)		71 (12.4%)	
Father with medical background		0.042		0.081		0.036		0.558		0.111
Yes	24 (22.6%)		8 (7.6%)		78(73.6%)		18 (16.9%)		22 (20.8%)	
No	261 (15.2%)		69 (4.0%)		1088 (63.5%)		255 (14.9%)		257 (15.0%)	
Mother with Medical background		<0.001		0.008		<0.001		0.675		0.123
Yes	34 (27.4%)		11 (8.9%)		100 (80.7%)		17(13.7%)		25 (20.2%)	
No	251 (14.8%)		66 (3.9%)		1066 (62.9%)		256 (15.1%)		254 (15.0%)	
Father’s education level	<0.001		0.020		<0.001		0.192		0.919	
No college	177 (13.2%)		48 (3.6%)		811 (60.5%)		210 (15.7%)		205 (15.3%)	
College	108 (22.6%)		29 (6.1%)		355 (74.3%)		63 (13.2%)		74 (15.5%)	
Mother’s education level	<0.001		0.007		<0.001		0.082		0.988	
No college	203 (14.0%)		52 (3.6%)		898 (62%)		228(15.8%)		222 (15.3%)	
College	82 (22.1%)		25 (6.8%)		268 (72.2%)		45 (12.1%)		57 (15.4%)	
Household income (Monthly)		0.014		0.015		0.383		0.177		0.040
High	232 (14.8%)		59 (3.8%)		997 (63.7%)		242 (15.5%)		251 (16.0%)	
Low	53 (20.9%)		18 (7.1%)		169 (66.5%)		31 (12.2%)		28 (11.0%)	
Hometown		<0.001		0.041		<0.001		0.263		0.016
Rural	108 (11.8%)		30 (3.3%)		523 (57.1%)		146 (15.9%)		159 (17.4%)	
Urban	177 (19.6%)		47 (5.2%)		643 (71.2%)		127 (14.1%)		120 (13.3%)	
KAP score		0.219		0.091		0.131		0.025		<0.001
0–7	15 (17.7%)		7 (8.2%)		49 (57.7%)		20 (23.5%)		25 (29.4%)	
8–12	168 (16.8%)		45 (4.5%)		659 (65.9%)		157 (15.7%)		169 (16.9%)	
13–15	102 (13.9%)		25 (3.4%)		458 (62.3%)		96 (13.1%)		85 (11.6%)	

Notes: SM: self medication; SMA: Self medication with antibiotics; B.A: Bachelor degree/ undergraduate degree; PhD: Doctor of Philosophy; KAP: knowledge, attitude, and practice.

**Table 5:** Logistic regression for the characteristics associated with the five behaviour outcomes among medical students in China in 2015.

	SM		Stock		Demand		SMA		Prophylaxis	
Factors	aOR (95% CI)	*p*	aOR (95% CI)	*p*	aOR (95% CI)	*p*	aOR (95% CI)	*p*	aOR (95% CI)	*p*
Sex										
Female			1.20 (1.04–1.56)	0.02						
Male			ref							
Age										
21–30					1.50 (1.00–2.20)	0.049				
31–40					1.50 (0.75–2.90)	0.264				
Father’s educational level										
Above college	1.50 (0.99–1.20)	0.052	1.60 (1.10–2.30)	0.007						
Less college	ref		ref							
Hometown	1.50 (1.10–2.00)	0.012								
Urban			1.60 (1.20–1.90)	<0.001					0.69 (0.50–0.94)	0.019
Rural	ref		ref						ref	
Mother with medical background										
No	0.62 (0.39–0.99)	0.049	0.53 (0.32–0.88)	0.014						
Yes	ref		ref							
KAP score										
8–12					0.58 (0.34–0.98)	0.045	0.52 (0.22–1.22)	0.132	0.52 (0.32–0.86)	0.011
12–15					0.46 (0.26–0.81)	0.007	0.37 (0.15–0.91)	0.031	0.35 (0.21–0.60)	<0.001
0–7					ref		ref		ref	

Note: Adjusted for university, age, sex, education level, hometown, household income, study year, parent’s education background, and whether parents with medical background. aOR: adjusted odd ratio. SM: Self-medication, SMA: Self-medication with antibiotics; KAP: knowledge, attitude, and practice.
